# Transcriptome Profiling Reveals Pro-Inflammatory Cytokines and Matrix Metalloproteinase Activation in Zika Virus Infected Human Umbilical Vein Endothelial Cells

**DOI:** 10.3389/fphar.2019.00642

**Published:** 2019-06-12

**Authors:** Svetlana Khaiboullina, Timsy Uppal, Konstatin Kletenkov, Stephen Charles St. Jeor, Ekaterina Garanina, Albert Rizvanov, Subhash C. Verma

**Affiliations:** ^1^Department of Microbiology and Immunology, University of Nevada, Reno, Reno, NV, United States; ^2^Department of Exploratory Research, Scientific and Educational Center of Pharmaceutics, Kazan Federal University, Kazan, Russia; ^3^Genequest LLC, Reno, NV, United States

**Keywords:** Zika virus, inflammation, cytokines, cell permeability, matrix metalloproteinase

## Abstract

The deformities in the newborns infected with Zika virus (ZIKV) present a new potential public health threat to the worldwide community. Although ZIKV infection is mainly asymptomatic in healthy adults, infection during pregnancy can cause microcephaly and other severe brain defects and potentially death of the fetus. The detailed mechanism of ZIKV-associated damage is still largely unknown; however, it is apparent that the virus crosses the placental barrier to reach the fetus. Endothelial cells are the key structural component of the placental barrier. Endothelium integrity as semi-permeable barrier is essential to control the molecules and leukocytes trafficking across the placenta. Damaged endothelium or disruption of adherens junctions could compromise endothelial barrier integrity causing leakage and inflammation. Endothelial cells are often targeted by viruses, including the members of the *Flaviviridae* family such as dengue virus (DENV) and West Nile virus (WNV); however, little is known about the effects of ZIKV infection of endothelial cell functions. Our transcriptomic data have demonstrated that the large number of cytokines is affected in ZIKV-infected endothelial cells, where significant changes in 13 and 11 cytokines were identified in cells infected with PRVABC59 and IBH30656 ZIKV strains, respectively. Importantly, these cytokines include chemokines attracting mononuclear leukocytes (monocytes and lymphocytes) as well as neutrophils. Additionally, changes in matrix metalloproteinase (MMPs) were detected in ZIKV-infected cells. Furthermore, we for the first time showed that ZIKV infection of human umbilical vein endothelial cells (HUVECs) increases endothelial permeability. We reason that increased endothelial permeability was due to apoptosis of endothelial cells caused by caspase-8 activation in ZIKV-infected cells.

## Introduction

Zika virus (ZIKV) is an approximately 11 kb positive-sense single-stranded enveloped RNA virus, classified into the *Flavivirus* genus within the *Flaviviridae* family, along with yellow fever virus (YFV), dengue virus (DENV), and West Nile virus (WNV). It is a rapidly emerging arbovirus (arthropod-borne virus), which was initially isolated from the serum of a febrile rhesus macaque in the Zika Forest of Uganda in 1947 and later on from the humans in Nigeria (Musso and Gubler, 2016). The virus, earlier thought to be restricted to the African and Asian continents, has now increased global attention due to its rapid spread throughout the Americas (Terzian et al., 2018) since its first detection in Brazil in May 2015.

Approximately 80% of ZIKV infections are asymptomatic, and the most common symptoms include fever, arthralgia, rash, myalgia, edema, vomiting, and non-purulent conjunctivitis (Moghadas et al., 2017). However, ZIKV infection in pregnant women has been linked to congenital microcephaly and other birth defects seen in neonates, such as placental insufficiency, fetal growth retardation, and fetal death (Chibueze et al., 2017; Ribeiro et al., 2018). ZIKV has also been linked to Guillain–Barré syndrome, a rare but serious auto-immune disorder (Parra et al., 2016). In humans, ZIKV is transmitted primarily *via* female mosquitoes, *Aedes aegypti* bite, through the skin of the infected host, followed by infection of permissive cells through specific receptors. Skin is thus believed to be the initial site of ZIKV replication, and from there, virus disseminates crossing blood–tissue barriers and could be detected in the brain, muscles, and placenta (Hamel et al., 2015; Aliota et al., 2016; Miner et al., 2016). Endothelial cells are the key elements of blood–brain barrier and a part of the placental blood barrier, which have been recognized as the site of flavivirus replication (Gowen et al., 2010; Beatty et al., 2015; Vervaeke et al., 2015). Earlier research studies on related flaviviruses, primarily DENV and YFV, showed that infected endothelial cells can produce inflammatory cytokines and chemokines, which further attract leukocytes to the site of virus propagation (Huang et al., 2000; Khaiboullina et al., 2005).

A recent study by Liu et al. (2016) reported that primary human endothelial cells are susceptible to productive infection by ZIKV. Specifically, both the African and South American ZIKV strains used in the study were found to efficiently infect and replicate in human endothelial cells, leading to the release of infectious virus. Conceptually, after its initial replication at the site of entry, ZIKV can spread *via* blood vessels while propagating in the infected endothelial cells (Liu et al., 2016). Recently, Szaba et al. (2018) have shown ZIKV antigens in embryonic endothelial cells and in the necrotic debris within the embryonic vasculature. These data strongly suggest the role of endothelial cells in propagation and dissemination of ZIKV to the fetus site.

Despite of intensive studies, our knowledge on alteration in transcriptional levels of cellular genes in ZIKV-infected endothelial cells remains limited. Also, the effect of ZIKV infection on the activation of cytokines in the endothelial cells is still poorly understood. The importance of cytokines for anti-viral protection and cellular immune defense activation has been well established (Dinarello, 2007). Mediators induced in the infected endothelial cells may contribute to the loss of vascular integrity and recruitment of leukocytes. Therefore, knowledge on cytokine activation in infected endothelial cell will help to better understand the role of host immune responses in pathogenesis of ZIKV.

Here, we show the transcriptional profiles of cellular genes following *de novo* infection of ZIKV in endothelial cells. Interestingly, changes in the transcriptional levels of genes differed between cells infected with South American and African strains of ZIKV. Several pathways were identified to be activated in HUVECs infected with ZIKV. Our data, for the first time, demonstrate ZIKV strain-specific activation of cytokines and matrix metalloproteinase (MMPs). Also, we report an upregulated production of an active form of caspase-8 in ZIKV-infected endothelial cells. Taken together, these findings explain the inflammation and destruction commonly seen in ZIKV-infected tissues.

## Materials and Methods

***Cell lines and Reagents***. Vero E6 cells were purchased from ATCC (American Type Culture Collection) and maintained in Dulbecco’s modified Eagle medium (DMEM) supplemented with 10% fetal bovine serum (FBS, Atlanta Biologicals), 2 mM L-glutamine, 25 U/ml penicillin, and 25 μg/ml streptomycin. Human umbilical vein endothelial cells (HUVECs) were purchased from and cultured in M200 medium enriched with 50× large vessel endothelial supplement (Salvesen and Dixit; Thermo Fisher Scientific). All cell lines were grown at 37°C in a humidified chamber supplemented with 5% CO_2_.

MMP inhibitor GM6001 (10 µM), GM6001 negative control (10 µM), and caspase-8 inhibitor, Ac-IETD-CHO (50 µM/ml), were purchased from Santa Cruz Biotechnology (Santa Cruz, CA).

***Infection of HUVECs***. ZIKV strains, PRVABC59 (Human/2015, Puerto Rico, South America) and IBH30656 (Human/1968, Nigeria), were obtained from ATCC (Manassas, VA) and subsequently propagated in Vero cells. Monolayer Vero cells were inoculated with each ZIKV strain for 2 h. Unattached virus was removed by washing with medium before incubating with fresh medium. Virions were collected after 7 days following the removal of cell debris and quantified by qPCR. HUVEC monolayers were incubated with each ZIKV (multiplicity of infection (MOI) ∼0.1) for 2 h. Virus was allowed to bind to the cells for 2 h at 37°C in a 5% CO_2_ atmosphere. Non-attached virus was removed by washing the cells with medium before incubating with the fresh medium. HUVECs were collected at indicated time points (3, 12, and 24 h) and stored at −80°C until used. Mock infection was carried out by incubation of HUVEC monolayers with supernatant from uninfected Vero cells for 2 h. In control experiments, ultraviolet (UV) light-inactivated ZIKV was used to infect HUVECs. ZIKV stocks were subjected to UV inactivation (1,200 µJ) in a UV Stratalinker 2400 (Stratagene).

***Plaque-forming assay for titrating ZIKV***. ZIKV titer was determined by plaque-forming assay as described previously (Khaiboullina et al., 2017). Briefly, infected Vero cells were overlaid with agarose (1%) containing DMEM medium supplemented with 10% FBS (HyClone, Logan, UT), 2 mM l-glutamine, 25 U/ml penicillin, and 25 μg/ml streptomycin. Seven days later, monolayers were fixed with 1% paraformaldehyde and stained with crystal violet (0.1%). All these assays were conducted under the biosafety level 2+ (BSL-2+) containment.

***Monocyte separation***. Cord blood buffy coats were obtained from the University of Colorado Cord Blood Bank, Aurora. Monocytes, CD14+ lymphocytes, were separated using Miltenyi magnetic bead separation kit (CD14 Microbeads; Miltenyi, Auburn, CA). Cells were rested overnight and cultured in DMEM medium supplemented with 10% FBS (HyClone, Logan, UT), 2 mM l-glutamine, 25 U/ml penicillin, and 25 μg/ml streptomycin.

Deidentified human cells were used in these assays, and all the experiments were done in accordance with guidelines of the University of Nevada, Reno. The Environmental and Biological Safety committee of the University of Nevada, Reno, approved the methods and techniques used in this study.

***Transwell migration assay***. Transwell system (5-µm pore size, Corning, Tewksbury, MA) was used to determine monocyte migration across HUVEC monolayer. HUVECs were seeded in the upper compartment and infected with PRVABC59 or IBH30656 ZIKV. Three days post-infection, monocytes (10^5^ cells/insert) were added into the upper compartment. Monocyte migration across the HUVEC monolayer was analyzed 24 h later by detecting monocytes in the lower compartment.

***RNA extraction and Next Generation Sequencing (NGS)***. Total RNA was extracted using Illustra RNAspin Mini kit (GE Healthcare). The RNA was quantified using NanoDrop UV spectrophotometer (ThermoFisher Scientific), and the quality of RNA was determined using Bioanalyzer (Agilent Technologies). The poly-A-containing mRNA was subjected to cDNA synthesis according to the TruSeq Stranded mRNA preparation guide (Illumina, San Diego, CA) to prepare the sequencing library. Validated and normalized libraries were diluted to a concentration of 4 nM, denatured with 0.2N NaOH, and then diluted to 20 pM with Hybridization buffer (HT1). The denatured and pooled libraries were pipetted onto a MiSeq Reagent Kit v3 and loaded on a HiSeq according to the manufacturer’s recommended procedure. The FastQ data generated by the HiSeq (Illumina, San Diego CA) was annotated, and the sequence reads were analyzed by CLC workbench 10.0.0 (Qiagen, Germantown, MD) for the detection of viral and cellular genes. Differential expressions of cellular gene expressions based on the RPKM (reads per kilobase of transcript per million mapped reads) were determined by comparing with mock-infected cells using RNA-seq analysis tool of CLC Workbench.

***Transwell permeability assay***. HUVEC monolayers were placed on the transwell polycarbonate filters (0.45-μm pore size; Costar, Brumath, France). Monolayers were infected with ZIKV for 72 h. Transmembrane diffusion of FITC-dextran (70 kDa; Sigma) was used to detect changes in permeability of HUVEC monolayers, as described by Lander et al. (2014). FITC-dextran (1 mg/ml) was added to the upper compartment of the transwell system. Aliquots (100 μl) of culture medium were collected from the lower chamber at 3-, 6-, 12-, 24-, and 48-h intervals. The fluorescence was measured using a spectrophotometer (Fluoroskan Ascent; ThermoFisher Scientific). OD values of culture medium in the lower chamber of ZIKV-infected cells were presented as percent change to that in mock-infected HUVECs, which were set as 100%.

***Quantitative PCR (qPCR)***. An aliquot of total RNA (40 ng) was used for synthesizing the cDNA (Superscript kit; ThermoFisher Scientific). Synthesized cDNA (1 μl for each target) was used for relative quantification of transcripts in a qPCR assay. ∆Ct values were calculated by normalizing with respective GAPDH Ct values, and the fold changes were calculated using ∆∆Ct method relative to the mock-infected control cells. The error bars represent standard deviation of three experiment replicates. Primer sequences are summarized in Table 1.

**Table 1:** Primer sequences.

Gene	Forward primer	Reverse primer
ZIKV	TTGGTCATGATACTGCTGATTGC	CCTTCCACAAAGTCCCTATTGC
Caspase-8	TTTGCTTGTCTCTCGGTGTC	CTCGAACAGTACGCCACACT
GAPDH	CCATGTTCGTCATGGGTGTGAACCA	CACGATACCAAAGTTGTCATGGA

***Western blotting***. Total protein was collected in 0.1% solution of sodium dodecyl sulfate (SDS) at 24 h post-infection and normalized using Bradford Protein Assay. Proteins were separated on a 9% polyacrylamide gel (BioRad, Hercules, CA), transferred onto nitrocellulose membrane, and blocked [5% non-fat milk in Tris-buffered saline (TBS) and 0.5% Tween 20] for 1 h at room temperature. Membranes were then incubated (18 h, 4°C) with the rabbit anti-ZIKV envelope protein polyclonal antibodies (1:5000; GTX133314; GeneTex, Irvine, CA), rat anti-caspase-8 (1:2,000, 645501; BioLegend, San Diego, CA), p-p38 mitogen-activated protein kinase (MAPK) (1:2,000, sc7973; Santa Cruz Biotechnology, Santa Cruz, CA), p38 MAPK (1:2,000, sc7149; Santa Cruz Biotechnology, Santa Cruz, CA), NF-κB (1:2,000, sc-8008; Santa Cruz Biotechnology, Santa Cruz, CA), Iκ-B (1:2,000; sc-1643, Santa Cruz Biotechnology, Santa Cruz, CA), and mouse anti-GAPDH (1:2,000; US Biological, Tustin, CA). The blots were washed (3×) with TBST and incubated with appropriate secondary antibodies conjugated with Alexa Fluor 680 (1:10,000, Molecular Probes, Carlsbad, CA). The membranes were scanned using the Odyssey scanner (Li-COR, Lincoln, NE).

***Cytokine detection***. The Bio-Plex human 21-Plex, 27-Plex, and 40-Plex and matrix metalloproteinase (MMP) 9-Plex (BioRad) were used for analyzing samples according to the manufacturer’s recommendations. Fifty microliters of the sample was used for determining cytokine concentration, and the collected data were analyzed using Luminex 200 analyzer with MasterPlex CT control software and MasterPlex QT analysis software (MiraiBio division of Hitachi Software San Francisco, CA, USA). Each Bioplex analysis was conducted in triplicates, and each experiment was repeated three times.

***Protein interaction network analysis:*** The Search Tool for the Retrieval of Interacting Genes/Proteins (STRING version 9.0) was used for analyzing interactions between cytokines with differential expression between ZIKV-infected and control HUVECs (Franceschini et al., 2013). STRING analysis was conducted using high confidence (score 0.7). Cluster analysis was conducted using k-means with a value of k = 3.

***Immunofluorescence analysis***. Cells were fixed (3:1 methanol/acetone) and stored at −80°C until use. Slides were permeabilized with 0.1% TritonX-100 for 30 min, washed (3×), and blocked (3% normal donkey serum, 0.5% BSA) for 60 min at room temperature. Monolayers were washed again (3×) and incubated with rabbit anti-ZIKV envelope polyclonal primary antibody (1:1,000; GeneTex, Irvine, CA), rat-anti VE cadherin antibody (1:100, 138101; Biolegend, San Diego, CA), or mouse anti-annexinV (1:100, sc-74438; Santa Cruz Biotechnology, Santa Cruz, CA) for 1 h at room temperature, followed by incubation with goat anti-rabbit Alexa Fluor 488, donkey anti-rat Alexa Fluor 488 (1:5000; Molecular Probe, Carlsbad, CA), or goal anti-mouse Alexa Fluor 488 secondary antibody for 1 h at room temperature in the dark, respectively. The nuclei were stained with TO-PRO-3 (ThermoFisher Scientific, Waltham, MA). Cells were examined by Carl Zeiss LSM 780 confocal laser-scanning microscope.

***Statistical analysis***. Statistical analyses were performed using Prism 6.0 software (Graphpad Inc.), and the p-values were calculated using two-tailed t tests. An asterisk represents statistical significance on the graphs. Pathway analysis was done using the Pathway Studio MammalPlus (Elsevier) package. Analytes which differ statistically between groups were used for enrichment analysis. For the enrichment analysis, only analytes with the adjusted p < 0.05 were selected. The Benjamini–Hochberg method was used to control the false recovery rate.

**Genebank accession number:** The RNA-seq data are submitted to Gene Expression Omnibus (accession number GSE103114) and can be accessed by the following link: https://www.ncbi.nlm.nih.gov/geo/query/acc.cgi?acc=GSE119232.

## Results

**Figure 1:**
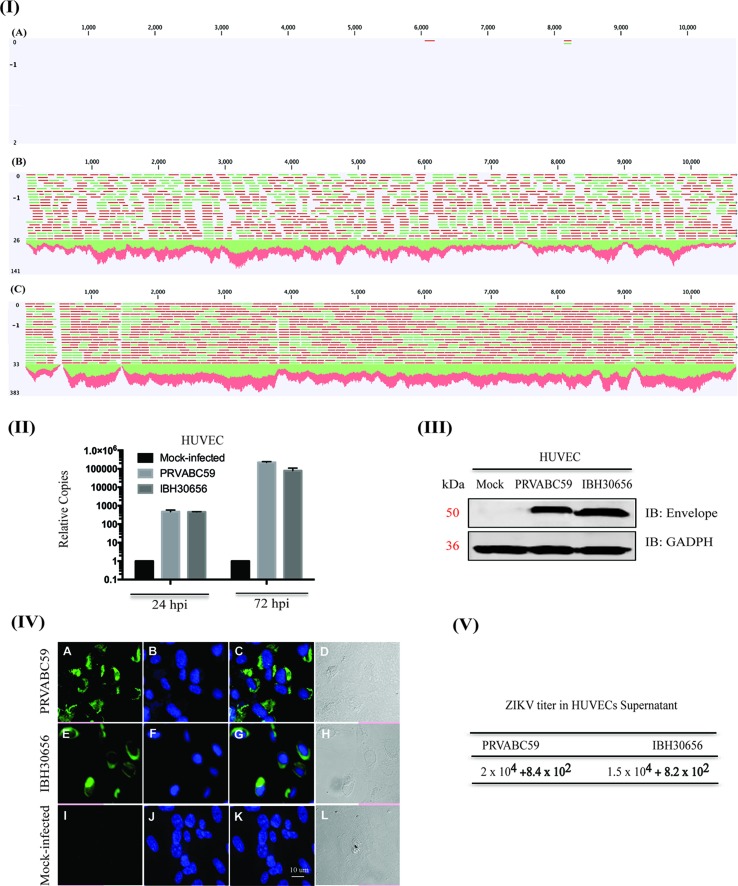
Zika virus (ZIKV) actively replicates in human umbilical vein endothelial cells (HUVECs). HUVECs were infected with PRVABC59 and IBH30656 ZIKV strains. ZIKV replication was detected by next-generation RNA-seq analysis (panel I), qPCR (panel II), Western blot (panel III), and immunofluorescence assay (panel IV) and plaque assay (panel V). **(I)** A: Mock-infected HUVECs (12 hpi); B: HUVECs infected with PRVABC59 ZIKV at 12 hpi; C: HUVECs infected with IBH30656 ZIKV at 12 hpi. **(II)** Total RNA was analyzed by qPCR targeting a region of the viral genome coding for the envelope protein. Relative copies of the viral genomes (ZIKV) were calculated using ∆∆Ct method. **(III)** Western blot analysis of envelope protein in ZIKV-infected HUVECs. Lane 1: Mock-infected HUVECs; lane 2: HUVECs infected with ZIKV strain PRVABC59; lane 3: HUVECs infected with ZIKV strain IBH30656. **(IV)** Immunofluorescence analysis of ZIKV envelope protein in the infected HUVECs. Images were captured using Carl Zeiss LSM780 confocal laser-scanning microscope. HUVECs infected with PRVABC59 ZIKV: A: envelope localization, B: nuclei; C: merge of A and B, D-DIC. HUVECs infected with IBH30656 ZIKV strain: E: envelope localization, F: nuclei; G: merge of E and F, H-DIC. Mock-infected HUVECs: I: envelope localization; J: nuclei; K: merge of I and J, L-DIC. Bar represents 10 µm size. **(V)** Plaque assay analysis of ZIKV replication in HUVECs. Supernatants were collected 72 h post-infection and used to determine infectious virus.

**ZIKV efficiently infects HUVECs**. HUVEC monolayers were infected with both the strains of ZIKV, PRVABC59 (South America) or IBH30656 (Nigeria), at a MOI of 0.1. HUVEC susceptibility to ZIKV infection and efficacy of virus replication was analyzed using next-generation sequencing of mRNA, real-time qPCR, Western blotting, and immunofluorescence assays. Mapping of the sequence reads with reference ZIKV genome revealed an accumulation of ZIKV transcripts in HUVECs, and a representative image of the mapped reads from 12 h post-infected samples is shown in **Figure 1**. The mock-infected HUVECs showed only one or two reads, as expected, while the ZIKV-infected cells showed a large number of reads (green and red lines representing reads) mapping to the viral genome, confirming that HUVECs were efficiently infected with both the strains of ZIKV (**Figure 1-I**; PRVABC59-panel B and IBH30656-panel C). To determine whether the incoming viral genome of ZIKV undergoes active replication, viral genome copies were quantified using qPCR at 24 and 72 h post-infection (hpi). Relative copies of ZIKV genome at these time points post-infection revealed an accumulation of ZIKV, confirming an active ZIKV replication in these cells (**Figure 1-II**). Detection of the viral envelope protein by Western blotting and immunofluorescence assays confirmed the transcriptional activation and expression of viral proteins following active replication of ZIKV (**Figure 1-III and IV**). The mock-infected cells lacking the band or signal for envelope protein confirmed the specificity of these assays (**Figure 1-III and IV**).

We also confirmed ZIKV replication in infected HUVECs by determining virions in the culture supernatant from 72 hpi cells through plaque-forming assay. Interestingly, both ZIKV strains, PRVABC59 and IBH30656, produced nearly similar number of plaques, 2 × 10^4^ and 1.5 × 10^4^, respectively, per ml of the culture supernatant (**Figure 1-V**).

**Figure 2:**
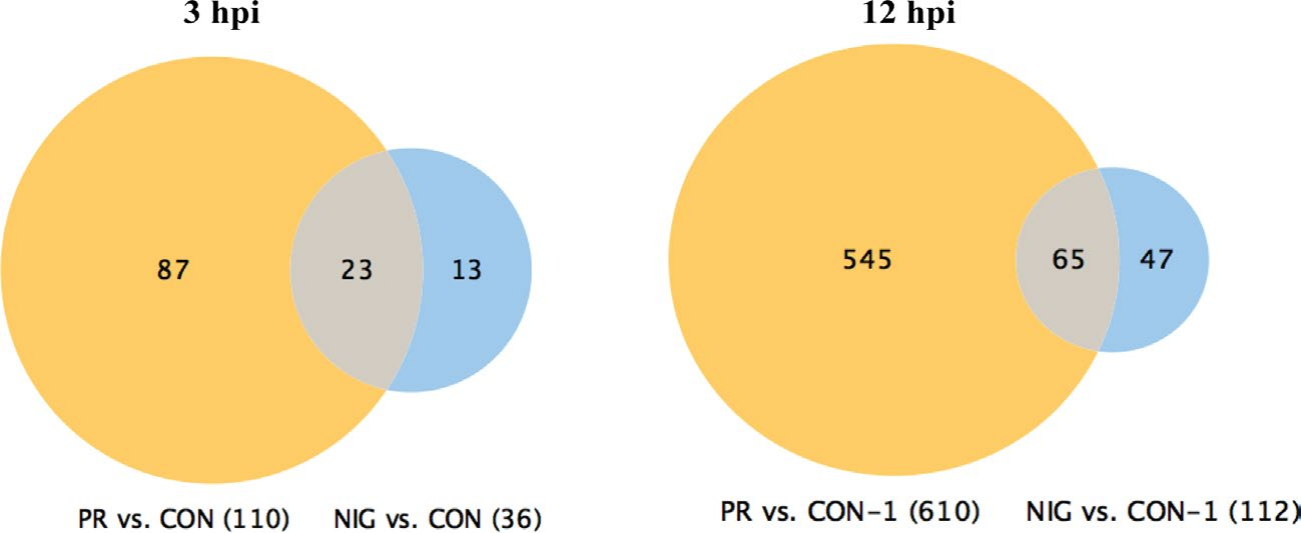
Venn diagram of genes affected in ZIKV-infected HUVECs. Changes in gene transcription were detected as early as 3 hpi, when transcriptional levels of 110 genes were affected in HUVECs infected with PRVABC59 ZIKV strain when compared to the mock-infected controls. Thirty-six genes were found differentially expressed at 3 hpi in HUVECs infected with IBH30656 ZIKV strain as compared to the control cells. Interestingly, 23 genes were affected by both ZIKV strains. At 12 hpi, 610 genes were affected by PRVABC59 ZIKV strain, while expression of only 112 was found changed in IBH30656 ZIKV-infected HUVECs. However, we found a group of genes (137 in PRVABC59 ZIKV and 326 in IBH30656 ZIKV), which were uniquely affected. Expression of 65 genes was affected by both strains.

**PRVABC59 ZIKV affects significantly higher number of cellular pathways as compared to the IBH30656 ZIKV**. Differential expression of cellular genes in ZIKV-infected HUVECs was analyzed by comparing with mock-infected control cells, and the genes with more than three-fold difference were selected for further analysis. Genes at early time post-infection (3 hpi) showed 110 differentially regulated genes in PRVABC59-infected HUVECs, whereas only 36 genes were differentially expressed in HUVECs infected with IBH30656 (**Figure 2**). Importantly, 23 genes were commonly affected by both the strains of ZIKV, as shown in a Venn diagram. Similarly, genes analyzed from 12 hpi cells showed 65 genes affected by both the strains of ZIKV. However, the total number of genes affected by PRVABC59 was significantly higher (610) as compared to those affected by IBH30656 (112) in infected HUVECs upon progression of infection.

### Identification of the Cellular Pathways Activated in ZIKV-Infected HUVECs Using Pathway Studio, MammalPlus

**Figure 3:**
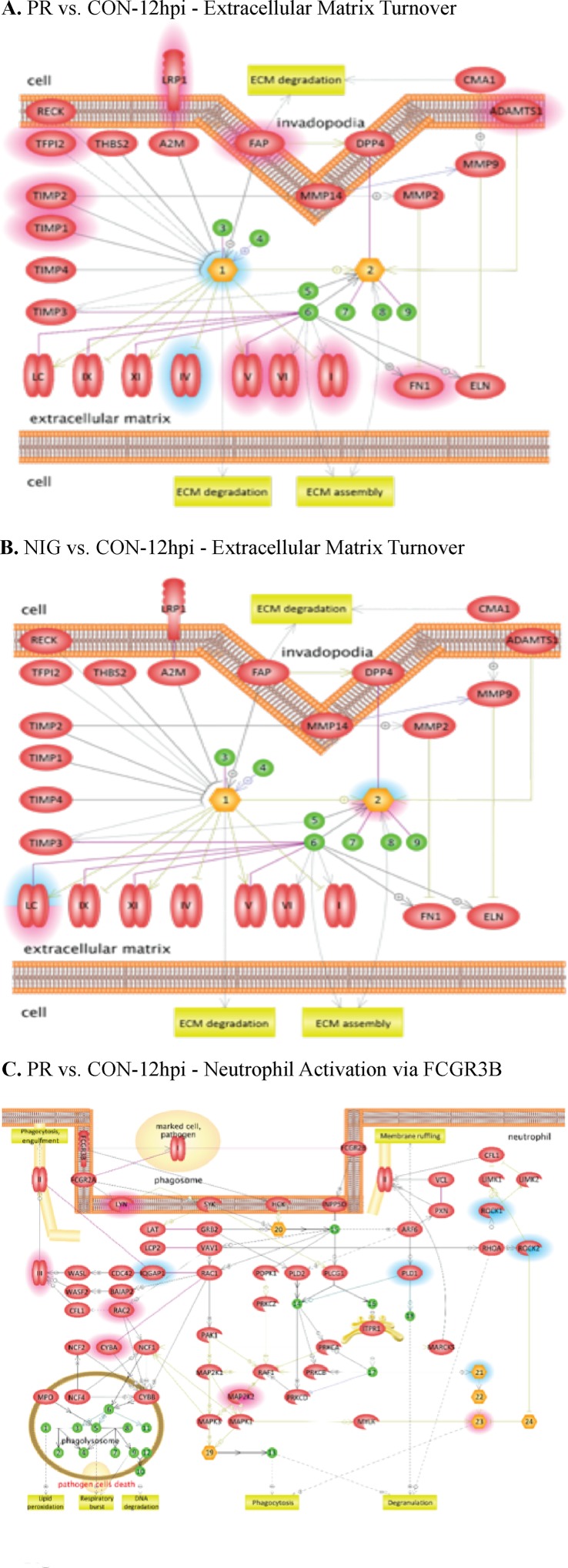
Pathway analysis of transcriptome data. Pathway analysis was done using the Pathway Studio MammalPlus (Elsevier) tool. Analytes that differ significantly between groups were used for enrichment analysis. Only pathways with p < 0.05 after correction for the multiple comparison were selected. Blue color highlights analytes, which were significantly lower in IBH30656 ZIKV-infected HUVECs. **(A)** PR vs.CON-12hpi – Extracellular matrix Turnover; **(B)** NIG vs.CON-12hpi – Extracellular matrix Turnover; **(C)** - PR vs. CON-12hpi -Neutrophil Activation via FCGR3B.

Differentially expressed cellular genes in these infected HUVECs were used for identifying pathways modulated by ZIKV. ZIKV infection affected multiple cellular genes and pathways at all the time points following infection including at 3 hpi. These pathways include leukocyte migration, leukocyte differentiation, platelet activation, focal cell junction assembly, and apoptosis. Interestingly, pathways involved in regulation of endothelial permeability, extracellular matrix turnover, platelets and neutrophil activation, as well as cytoskeleton reorganization were affected in ZIKV-infected HUVECs. Among these, PRVABC59 ZIKV infection led to an increase in the expression of cytochrome c, while IBH30656 infection resulted in a decreased level of cytochrome c (**Figure 3**).

**Figure 4:**
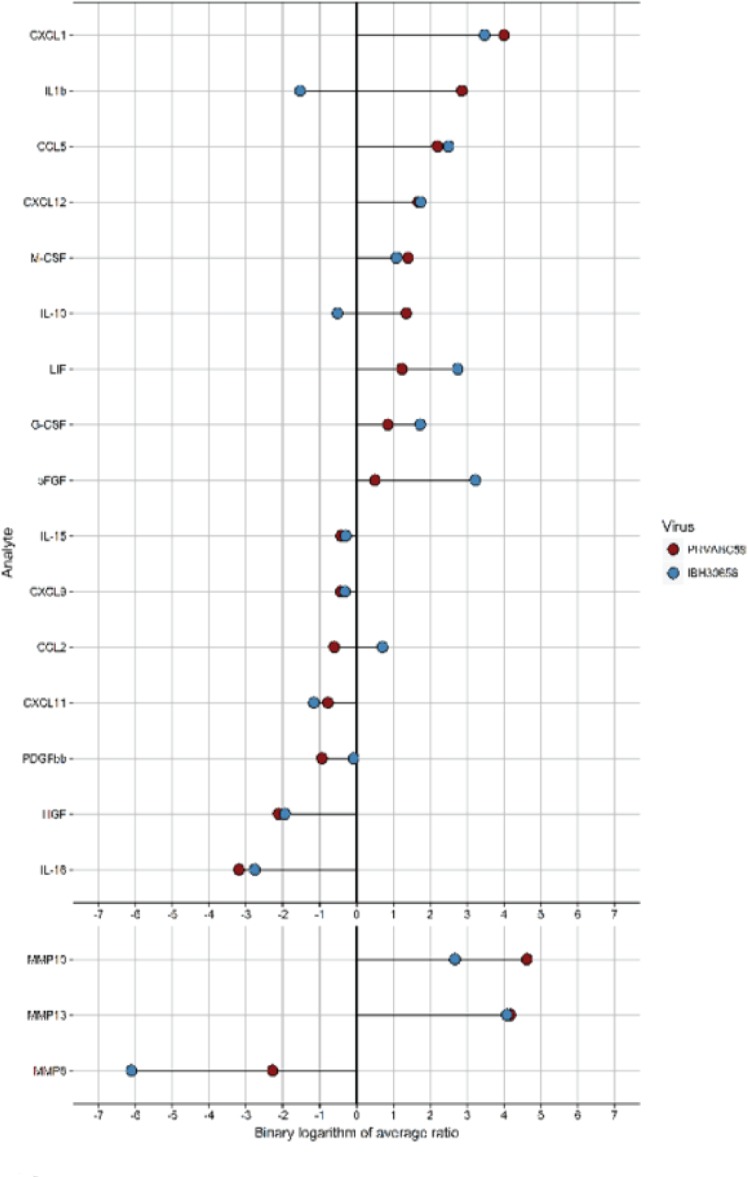
Analysis of cytokines and MMP activation in ZIKV-infected HUVEC multiplex data. Culture supernatant (50 µl) was used to detect the level of 58 analytes (Bio-Plex human 21-Plex, 27-Plex, 40-Plex; BioRad) and 9 analytes (MMP 9-Plex; BioRad), respectively. Significantly different analytes (between infected and mock-infected groups) were used to generate the diagram. For every analyte, the ratio of cytokine median level in each ZIKV-infected HUVECs to that in mock-infected control was calculated. The binary logarithm of these values was used for sorting of analytes and generation of “lollypop” diagram with “ggplot2” package.

**Cytokines and MMPs activation in ZIKV-infected HUVECs.** Cytokine activation has been previously demonstrated in flavivirus-infected HUVECs (Lin et al., 2002; Khaiboullina et al., 2005). Therefore, we sought to determine whether ZIKV infection activates cytokine production in HUVECs. Culture supernatants from the mock-infected HUVECs as well as cells infected with either ZIKV strains were collected at 24 hpi and used for determining the levels of cytokines (**Table 2**). Out of 58 cytokines tested, the levels of 13 cytokines (IL-1β, IL-10, IL-15, IL-16, CCL5, G-CSF, HGF, LIF, MCSF, PDGFbb, CXCL1, CXCL9, and CXCL12) were affected in HUVECs infected with PRVABC59 ZIKV, while 11 cytokines (IL-15, CCL2, CCL5, bFGF, G-CSF, HGF, LIF, MCSF, CXCL1, CXCL11, and CXCL12) were altered in IBH30656 ZIKV-infected HUVECs as compared to the mock-infected controls (**Table 2**). Among these, eight cytokines (IL-1β, IL10, CCL5, G-CSF, CSF, CXCL1, and CXCL12) were upregulated and five (IL-15, IL-16, HGH, PDGFbb, and CXCL9) were downregulated in PRVABC59-infected HUVECs compared to the mock-infected HUVECs (**Figure 4**). Out of the 11 cytokines affected in IBH30656-infected HUVECs, 8 cytokines were higher (CCL2, CCL5, bFGF, G-CSF, LIF, M-CSF, CXCL1, and CXCL12), while 3 (IL-15, HGH, and CCXL11) were lower than the mock-infected control cells. Cytokines IL-15, CCL5, HGF, LIF, M-CSF, CXCL1, and CXCL12 were commonly upregulated by PRVABC59 and IBH30656. Interestingly, some cytokines were expressed in HUVECs infected with each ZIKV strain. IL-1β, IL-10, IL-16, PDGFbb, and CXCL9 were altered only in PRVABC59-infected HUVECs as compared to mock-infected control, while only three cytokines, namely, CCL2, bFGF, and CXCL11, were altered in IBH30656-infected HUVECs.

**Table 2:** Cytokine and metalloproteinase activation in HUVECs infected with PRVABC59 ZIKV and IBH30656 ZIKV.

Cytokine	Mock-infected (pg/ml)	PRVABC59 (pg/ml)	IBH30656 (pg/ml)
IL1b	104.5 ± 38.3	757.2 ± 67.7, p < 0.003	36.2 ± 2.4
IL-10	676.4 ± 103.2	1,721.4 ± 342, p < 0.04	472.8 ± 2.7
IL-15	616.8 ± 10.6	461.0 ± 31.7, p < 0.009	502.5 ± 24.9, p < 0.01
IL-16	714.1.2 ± 209.2	78.3 ± 18.8, p < 0.055	105.3 ± 30.8
CCL2	4,213.5 ± 957.2	2,766.6 ± 394.9	6,858.1 ± 87.4, p < 0.051
CCL5	157.1 ± 17.2	717.9 ± 142.8, p < 0.01	886.0 ± 82.2, p < 0.002
bFGF	287.3 ± 22.1	406.1 ± 71.1	2,679.8 ± 188.6, p < 0.02
G-CSF	458.3 ± 12.3	825.7 ± 48.3, p < 0.001	1,519.8 ± 312.2, p < 0.02
HGF	135.5 ± 27.5	31.2 ± 10.9, p < 0.01	35.3 ± 11.4, p < 0.04
LIF	6.1 ± 0.1	14.3 ± 3.1, p < 0.05	40.8 ± 11.0, p < 0.03
M-CSF	7.4 ± 0.9	19.5 ± 5.4, p < 0.01	15.7 ± 0.7, p < 0.001
PDGFbb	2,689.8 ± 198.1	1,403.9 ± 391.1, p < 0.04	2,545.8 ± 652.9
CXCL1	174.8 ± 76.1	2,802.1 ± 817.4, p < 0.02	1,941.3 ± 503.6, p < 0.02
CXCL9	6.6 ± 0.4	4.9 ± 0.2, p < 0.03	5.3 ± 0.2
CXCL11	146.7 ± 8.9	85.6 ± 46.8	65.7 ± 21.9, p < 0.02
CXCL12	16.0 ± 2.8	51.1 ± 12.0, p < 0.05	53.4 ± 2.7, p < 0.0009

Cytokine activation and endothelial monolayer permeability is regulated by matrix metalloproteinases (MMPs), which act by degrading the extracellular matrix to facilitate the movement of molecules and cells across the blood–tissue barrier (Lakhan et al., 2013). Since changes in MMPs have been demonstrated in HUVECs infected with *Flaviviridae*, we sought to determine whether ZIKV infection will affect secreted MMP levels (**Table 3**). Indeed, the levels of secreted MMP8, MMP10, and MMP13 were altered in HUVECs infected with both the strains of ZIKV. Interestingly, the levels of MMP8 were reduced, while MMP10 and MMP13 levels were increased in ZIKV-infected as compared to the mock-infected cells.

**Table 3:** MMPs activation in HUVECs infected with PRVABC59 ZIKV and IBH30656 ZIKV.

MMP	Mock-infected (pg/ml)	PRVABC59 (pg/ml)	IBH30656 (pg/ml)
MMP8	1,246.9 ± 86.5	257.4 ± 38.8, p < 0.0005	18.1 ± 0.4, p < 0.0001
MMP10	607.7 ± 131.2	14,937.3 ± 678.4, p < 0.0001	3,865.1 ± 885.4, p < 0.02
MMP13	10.5 ± 0.3	189.7 ± 40.8, p < 0.01	177.6 ± 62.6, p < 0.05

**Figure 5:**
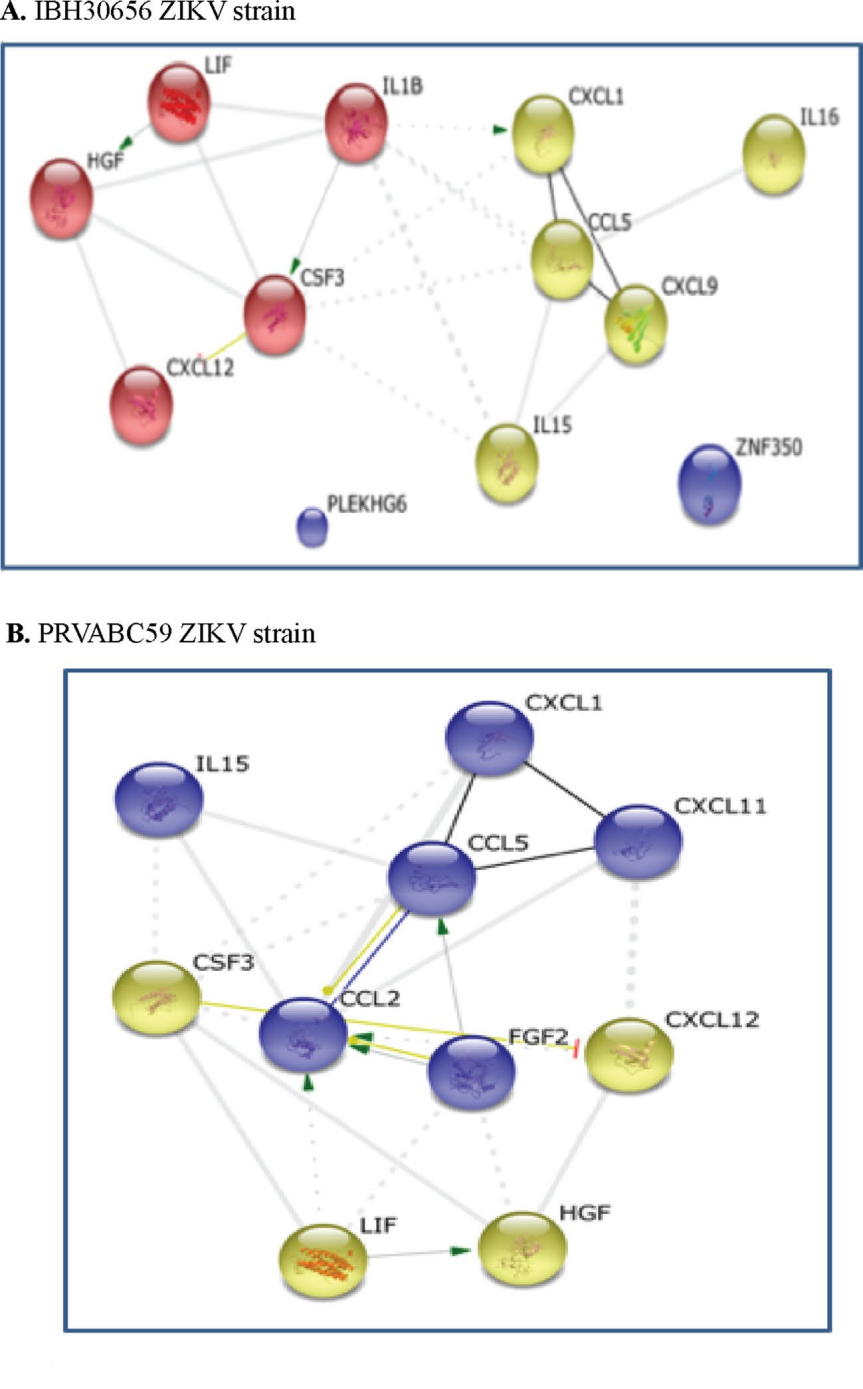
Interaction between cytokines in ZIKV-infected HUVECs: green, activation; blue, binding; black, reaction; thicker the line = stronger interaction; String 9.0 (http://string-db.org) high confidence 0.7 (circled are clusters k means 3). **(A)** 1BH30656 ZIKV strain, **(B)** PRVABC59 ZIKV strain.

**String analysis**. STRING analysis generates a protein interaction profile using different sources including interaction databases, text mining, and genetic interactions (Szklarczyk et al., 2015). Cytokines having significantly altered expression in HUVECs due to ZIKV infection were used as an input to the STRING tool. STRING provides different viewing options including action, evidence, and confidence. STRING analysis identified two clusters in HUVECs infected with both the strains of ZIKV (**Figure 5**). The upregulated cytokines in IBH30656-infected HUVECs were in two clusters: G-CSF, HGF, LIF, and CXCL12 in the first cluster, and IL-15, CCL2, CCL5, CXCL1, CXCL11, and FGFb in the second cluster (**Figure 5A**). In PRVABC59-infected HUVECs, one cluster contained IL-1β, G-CSF, HGF, LIF, and CXCL12, while IL-15, IL-16, CCL5, CXCL1, and CXCL9 were in another cluster (**Figure 5B**). In STRING analysis connection between these two clusters, it appears that IL-1β regulates the expression of GM-CSF and CXCL1, while bFGF activates CCL2 and CCL5.

**Figure 6:**
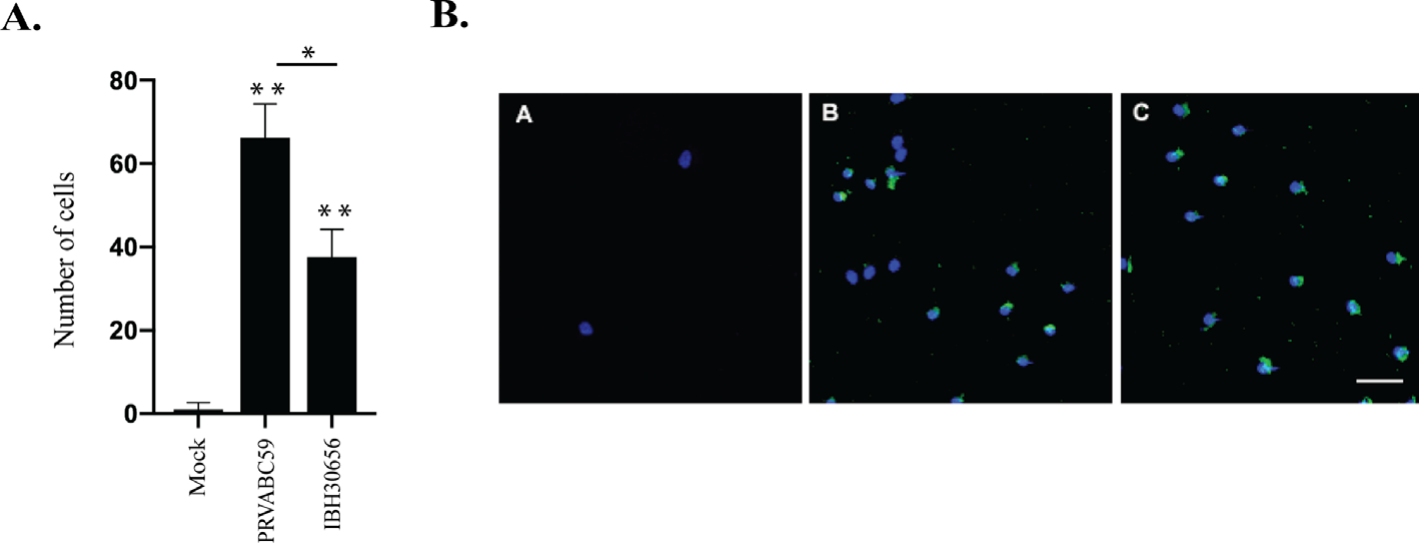
Monocyte migration across ZIKV-infected endothelial monolayer. HUVECs were seeded in the upper compartment of the Boyden chamber and infected with PRVBC59 or IBH30656 ZIKV (MOI 0.1). Three days later, monocytes (10^5^ cells/inset) were added in the upper compartment. Migrated monocytes were analyzed 24 h later in the lower compartment. Experiments were repeated four times. **(A)** Monocyte count in the lower chamber. Monocytes were counted in five separate fields; *p < 0.03, **p < 0.0001 by paired t test. Data shown are the mean ± Sterr of four independent experiments. **(B)** Immunofluorescence analysis of monocytes in the lower chamber: A: mock-infected control; B: PRVBC59 ZIKV infected; C: IBH30656 ZIKV infected.

**ZIKV-induced trans-endothelial migration of monocytes.** MMPs and cytokines/chemokines play an important role in regulation of leukocyte migration across endothelium (Gerhardt and Ley, 2015). We sought to determine whether infection of HUVECs with ZIKV will enhance monocyte cross endothelial migration. We infected HUVECs with PRVABC59 and IBH30656 ZIKV at MOI of 0.1 and determined migrating monocytes, which showed a significant number of monocytes that migrated across the ZIKV-infected HUVEC monolayer as compared to the mock-infected control HUVEC monolayer (**Figure 6A**). Importantly, these migrated monocytes were ZIKV antigen positive, suggesting that they could contribute to the dissemination of ZIKV across the endothelial barrier (**Figure 6B**).

**Figure 7:**
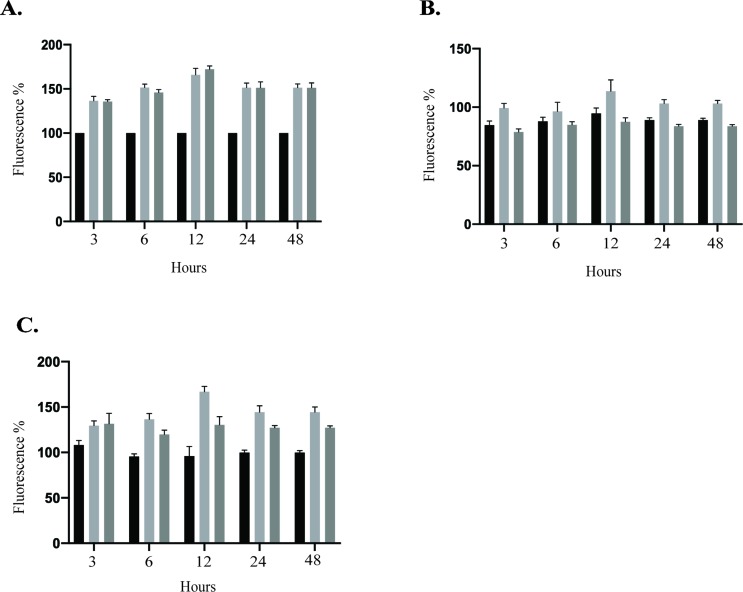
Transwell permeability assay. HUVECs were seeded onto Transwell inserts and infected with PRVABC59 or IBH30656 ZIKV (MOI 0.1) or mock. FITC-dextran was added into the upper compartment of the Transwell system. Aliquots of culture medium from the lower chamber were collected at selected time points to determine the presence of dextran molecule. Data are presented as a percent change in permeability as compared to mock-infected control. MMPs were inhibited using GM6001 (10 µM). Also, GM6001 negative control (10 µM) was used. **(A)** FITC-dextran permeability of HUVECs infected with PRVABC59 or IBH30656 ZIKV; **(B)** FITC-dextran permeability of HUVECs infected with PRVABC59 or IBH30656 ZIKV and treated with GM6001; **(C)** FITC-dextran permeability of HUVECs infected with PRVABC59 or IBH30656 ZIKV and treated with GM6001, negative control. ***p < 0.0001 by paired t test. Data shown are the mean ± Sterr of three independent experiments.

**ZIKV increased HUVEC permeability.** Several members of the *Flaviviridae* family are known to target endothelial cells and affect vascular endothelium permeability (Gowen et al., 2010; Beatty et al., 2015; Vervaeke et al., 2015). Change in permeability could lead to local edema and inflammation, which are hemorrhages and leukocyte extravasation (Nourshargh and Alon, 2014; Chanthick et al., 2018). Therefore, we sought to determine whether ZIKV infection disturbs the permeability of HUVEC monolayer *in vitro* using modified Boyden chamber system. Following infection of HUVECs with PRVABC59 or IBH30656 ZIKV (MOI 0.1), FITC-dextran was added into the upper compartment of the Transwell system. Aliquots of culture medium from the lower chamber were collected at selected time points to determine the presence of dextran molecule. Significantly higher amount of the 70-kDa FITC-dextran was detected in the lower compartment of ZIKV-infected endothelial monolayers as compared to mock-infected control cells (**Figure 7A**). Importantly, both ZIKV strains caused increased dextran permeability.

Since MMPs are implicated in pathogenesis due to an increased endothelial monolayer permeability (Alexander and Elrod, 2002), we sought to determine whether inhibition of MMPs will restore the permeability of ZIKV-infected HUVEC monolayer. HUVECs were infected with PRVABC59 or IBH30656 ZIKV (MOI 0.1) followed by changing the medium and replacing with fresh medium supplemented with MMP inhibitor GM6001 (10 µM) or GM6001 negative control (10 µM). HUVEC monolayer permeability was determined by FITC-dextran detection in the lower compartment of the Transwell system. Interestingly, inhibition of MMPs led to a restoration of ZIKV-mediated cellular permeability almost to a level in uninfected control cells (**Figure 7B**). MMP negative control did not affect the endothelial monolayer permeability and failed to restore permeability in ZIKV-infected cells (**Figure 7C**).

**Figure 8:**
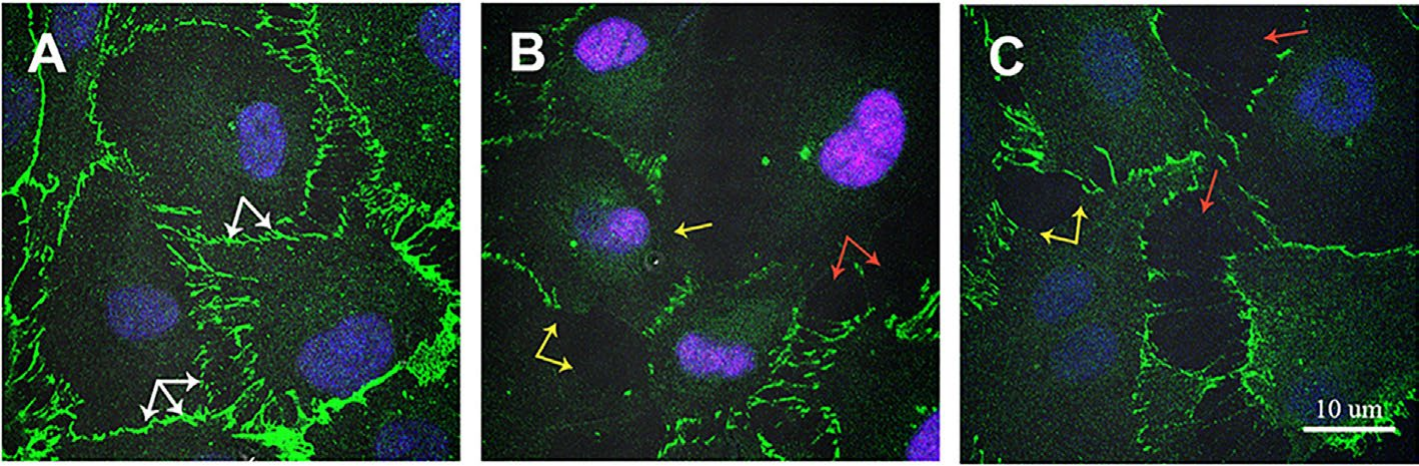
Immunofluorescence analysis of VE cadherin expression in ZIKV-infected HUVECs. HUVECs were infected with PRVABC59 and IBH30656 ZIKV strains for 72 h; monolayers were fixed and probed with rat anti-VE cadherin antibody. Nuclei were stained with TO-PRO-3. Images were captured using Carl Zeiss LSM 780 confocal laser-scanning microscope. **(A)** mock-infected control HUVECs; **(B)** HUVECs infected with PRVABC59 ZIKV; **(C)** HUVECs infected with IBH30656 ZIKV. White arrow: VE cadherin expression in control HUVECs; yellow arrow: decreased VE cadherin expression in ZIKV-infected HUVECs; red arrow: free of VE cadherin spaces between ZIKV-infected HUVECs. Bar represents 10 µm size.

**ZIKV infection changed VE-cadherin expression.** VE-cadherin plays an important role in regulating endothelial permeability (Gavard, 2013). Therefore, we sought to determine whether changes in permeability could be explained by ZIKV affecting VE-cadherin expression (**Figure 8**). Strong VE cadherin expression was demonstrated in mock-infected HUVECs. VE cadherin accumulation was detected as continuous lines around each HUVEC cell (**Figure 8A**, white arrow). However, VE cadherin expression was changed in ZIKV-infected HUVECs, where its expression was lower in some areas of endothelial cells (**Figure 8B and C**, yellow arrow). Also, open spaces free of VE cadherin were detected in ZIKV-infected HUVECs (**Figure 8B and C**, red arrow).

**Figure 9:**
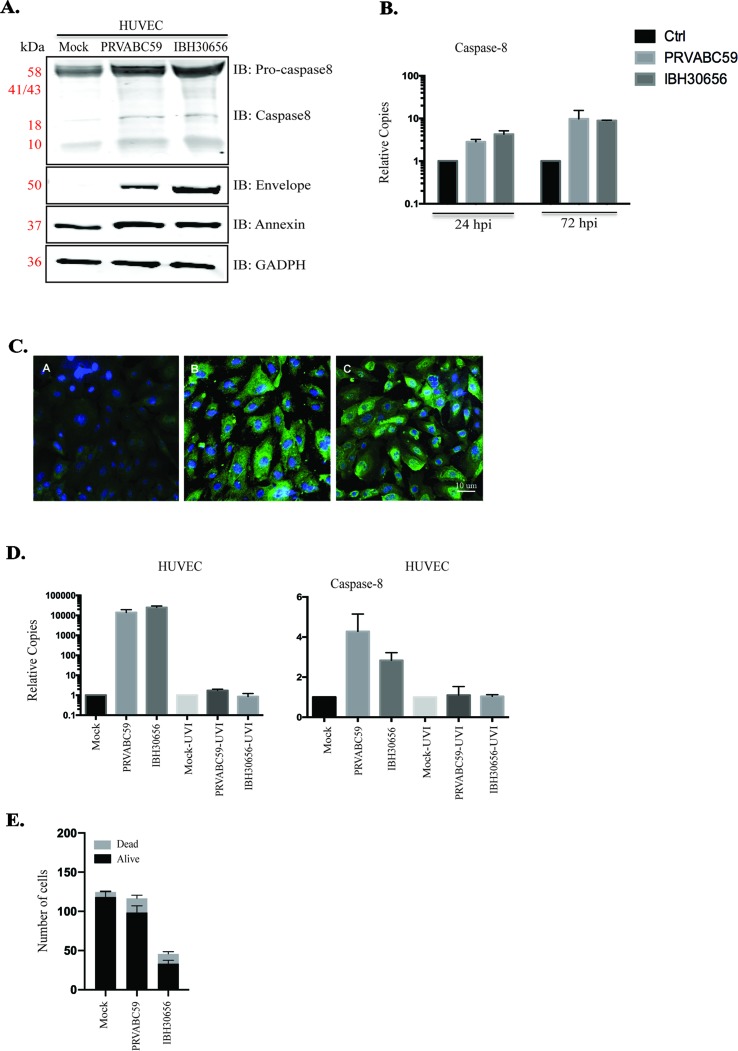
Caspase-8 and annexinV activation in ZIKV-infected HUVECs. ZIKV replication was analyzed using Western blot (panel A) and qPCR (panel B). **(A)** Western blot analysis of envelop protein, caspase-8, and annexinV in ZIKV-infected HUVECs. HUVECs were infected with PRVABC59 and IBH30656 ZIKV. At 72 hpi, total proteins were collected and used for the detection of envelop protein, caspase-8, and annexinV. GAPDH was detected as the loading control. Lane 1: mock-infected HUVECs, lane 2: HUVECs infected with ZIKV strain PRVABC59; lane 3: HUVECs infected with ZIKV strain IBH30656. **(B)** qPCR analysis of virus transcript accumulation and caspase-8 transcription activation in ZIKV-infected HUVECs. Relative copies of each transcript were calculated using ∆∆Ct method. **(C)** IFA analysis of annexinV expression in ZIKV-infected HUVECs. A: mock-infected control; B: ZIKV strain PRVABC59; C: ZIKV strain IBH30656. Bar represents 10 µm size. **(D)** Effect of ZIKV replication on caspase-8 expression. UV-inactivated and replication-competent ZIKV PRVABC59 and IBH30656 were used to infect HUVECs. Total RNA was collected at 72 hpi and analyzed using qPCR. Relative copies of the viral genomes (ZIKV) were calculated using ∆∆Ct method. **(E)** Effect of ZIKV infection on cell vitality: mock-infected and ZIKV-infected HUVECs (IBH30656 and PRVABC59 strains) were counted using trypan blue. Experiments were performed in triplicate for three independent times.

**ZIKV-induced caspase-8 in HUVECs**. Increased endothelial permeability could be due to the ZIKV-induced endothelial cell death, which has been documented in cells infected with DENV, WNV, and Japanese Encephalitis virus, other members of *Flaviviridae* (Ghosh Roy et al., 2014). Our pathway data analysis indicates cytochrome c mobilization in ZIKV-infected HUVECs. The mitochondria activation may lead to an upregulation of caspase-8, which plays important role in the initiation of apoptosis (Elmore, 2007). Caspase-8 is also known to activate caspase-3, a main executional caspase (Elmore, 2007). Our data showed an increased level of full-length caspase-8 (Procaspase-8) and the release of an active form of caspase-8 in both ZIKV-infected cells (**Figure 9A**). Viral replication was confirmed by expression of ZIKV envelope protein (**Figure 9A**). An increase in the transcriptional levels of caspase-8 was determined in ZIKV-infected HUVECs (**Figure 9B**). Also, it appears that ZIKV replication is required for caspase-8 activation, as increased level of caspase-8 transcripts was found only in HUVECs infected with infectious virus, while these transcripts were absent in cells infected with UV-inactivated ZIKV (**Figure 9D**).

Since caspase-8 is an executive caspase in the apoptotic pathway, we sought to determine whether ZIKV triggers cell death. Apoptosis was evaluated by the detection of annexinV expression in ZIKV-infected cells. Increased annexinV expression was demonstrated in ZIKV-infected HUVECs using Western blot (**Figure 9A**) and immunofluorescence assays (**Figure 9C**). To determine the effects of ZIKV on HUVEC cell growth and viability, 4 × 10^5^/well cells were placed into the six-well plate before infection with PRVABC59 or IBH30656 at MOI of 0.1. Cells were trypsinized after 72 hpi and counted using trypan blue (**Figure 9D**). The number of HUVECs in uninfected controls was 1.18 × 10^6^ ± 7.7 × 10^4^, while the number of live cells was significantly reduced in sets infected with IBH30656 ZIKV (9.8 × 10^5^ ± 8.5 × 10^4^; p < 0.1) or PRVABC59 (3.3 × 10^5^ ± 4.4 × 10^4^; p < 0.0001) (**Figure 9E**).

**Figure 10:**
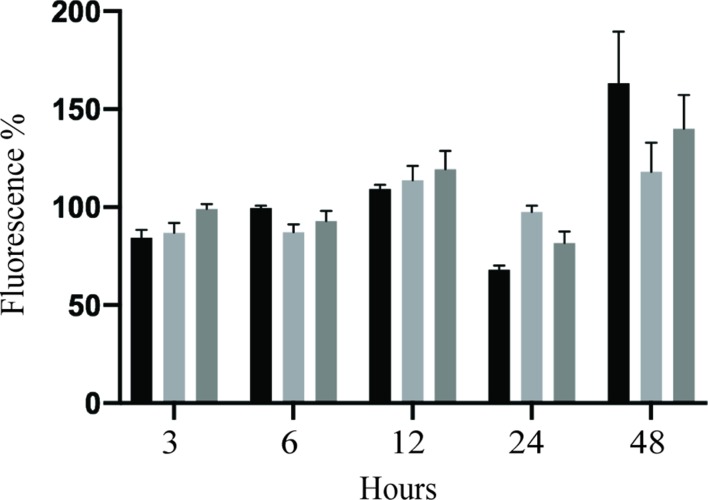
Effect of caspase-8 inhibition on transwell permeability of ZIKV-infected HUVECs. HUVECs were seeded onto Transwell inserts and infected with IBH30656, PRVABC59 ZIKV (MOI 0.1), or mock. FITC-dextran was added into the upper compartment of the Transwell system. Aliquots of culture medium from the lower chamber were collected at selected time points to determine the presence of dextran molecule. Data are presented as a percent change in permeability as compared to the mock-infected control. Caspase-8 was inhibited using Ac-IETD-CHO (50 µM).

**Caspase-8 activation linked cell death could contribute to HUVEC monolayer permeability.** To address this assumption, HUVEC monolayer permeability was analyzed *in vitro* using modified Boyden chamber system. HUVEC monolayers were infected with PRVABC59 or IBH30656 ZIKV (MOI 0.1) for 1 h, virus inoculum was removed, and fresh medium supplemented with Ac-IETD-CHO (50 µM), a caspase-8 inhibitor, was added. FITC-dextran was applied into the upper compartment of the Transwell system, and aliquots of culture medium from the lower chamber were collected at selected time points. Significantly higher amount of the 70 kDa FITC-dextran was detected in the lower compartment of ZIKV-infected HUVEC monolayers as compared to mock-infected controls (**Figure 10**). Inhibition of caspase-8 decreased permeability of HUVECs infected with PRVABC59 or IBH30656 ZIKV. These data suggest that activation of caspase-8 alters the endothelial monolayer integrity of ZIKV-infected cells.

**Figure 11:**
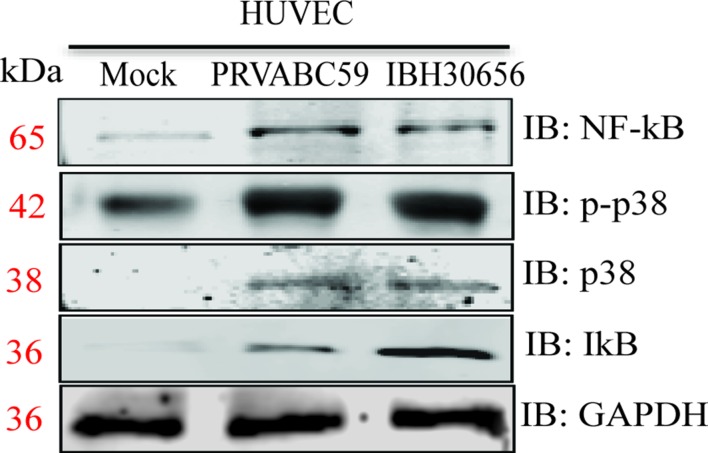
Activation of p-p38 MAPK, p38 MAPK, and NF-κB kinases in ZIKV-infected HUVECs. HUVECs were infected with PRVABC59 and IBH30656 strains of ZIKV. At 72 hpi, total proteins were collected and used to determine the level of p-p38 MAPK, p38 MAPK, and NF-κB kinases. GAPDH was detected as loading control.

**ZIKV activates mitogen-activated protein kinase p38 and NF-κB kinases**. MAPK p38 and NF-κB are shown to be activated in cells infected with *Flaviviruses* (Marianneau et al., 1997; Kesson and King, 2001). Activation of these kinases is essential for upregulation of cytokines and matrix metalloproteinases (Prickett and Brautigan, 2007; Lawrence, 2009). Therefore, we sought to determine whether ZIKV infection also upregulates these kinases. HUVECs were infected with PRVABC59 (South America) or IBH30656 (Nigeria) at a MOI 0.1. Proteins were collected at 72 hpi and used for Western blot analysis of p38 and NF-κB expression (**Figure 11**). Both ZIKV increased expression of p38 MAPK and NF-κB kinases. Increased level of IκB was demonstrated in ZIKV-infected cells, suggesting its dissociation from NF-κB. Also, increased expression of phosphorylated p38 kinase was found in ZIKV-infected cells.

## Discussion

ZIKV is a member of the Flavivirus family, which was recently shown to be associated with severe defects in newborns, microcephaly (Campos et al., 2015). Although infection is often asymptomatic and rarely presents with clinical symptoms, the newborn brain is targeted by the virus, causing tissue damage and inflammation (Chibueze et al., 2017; Moghadas et al., 2017). It appears that after initial replication at the site of entry, virus disseminates, reaching tissues and organs including placenta. Placenta is a complex barrier, where endothelial cells play central role in regulating the transfer of materials between the mother and the fetus (Griffiths and Campbell, 2015). Endothelium plays an important role in regulating barrier permeability and protecting fetus from pathogens (Pang et al., 2017). However, viruses targeting endothelial cells could compromise endothelial monolayer integrity and consequently can cross the organ barrier. Our data suggest that endothelial cells are susceptible to ZIKV infection and cause cytokine activation and increased endothelial monolayer permeability, which could contribute to pathogenesis of ZIKV infection.

Endothelial cells are susceptible to ZIKV infection (Papa et al., 2017; Roach and Alcendor, 2017; Peng et al., 2018), where virus replication was shown in human brain microvascular endothelial cells (HBMECs), retinal endothelial cells (RECs), and HUVECs. ZIKV infection of HBMECs and RECs led to activation of several proinflammatory cytokines including IL-6, CCL1, and CCL5 (Papa et al., 2017; Roach and Alcendor, 2017). To expand our understanding on inflammatory response in ZIKV-infected endothelial cells, we sought to determine changes in cytokines and chemokines in ZIKV-infected endothelial cells. Additionally, ZIKV effect on MMPs in HUVECs was investigated.

We show that ZIKV infection activates proinflammatory (IL-1β) cytokines as well as chemokines (CCL2, CCL5, GM-CSF, G-CSF, CXCL1, and CXCL12) in the endothelial cells. The cytokine upregulation could be explained by increased expression of p38 MAPK and NF-κB kinases in ZIKV-infected cells. These kinases are major regulators of cell responses to various stimuli including cytokine activation (Prickett and Brautigan, 2007; Lawrence, 2009). Interestingly, the cytokines activated in HUVECs suggests that multiple subsets of leukocytes could be attracted to the site of infection including mononuclear leukocytes (monocytes and lymphocytes) as well as granulocytes (neutrophils). Additionally, we found that the activation of cytokines in HUVECs was ZIKV strain dependent as 13 cytokines were affected in PRVABC59-infected cells as compared to 11 cytokines in IBH30656-infected cells. It could suggest that ZIKV infection promotes mononuclear leukocytes and neutrophil chemotaxis across the endothelial barrier. Recently, ZIKV-positive monocytes were demonstrated in the blood of infected individuals (Lum et al., 2017). We have also shown that monocytes are susceptible to ZIKV infection and virus replication (Khaiboullina et al., 2017). Therefore, we suggest that ZIKV-infected endothelial cells attract monocytes carrying viral antigens and promote their migration into the tissue.

It is striking that the level of IL-1β was upregulated in HUVECs infected with PRVABC59 ZIKV, and it remained unaffected in cells infected with IBH30656 ZIKV. IL-1β is a product of activated inflammasomes (Netea et al., 2015). Inflammasomes are complex polymers assembled when pathogen recognition receptors (PRRs) are activated (Ferreri et al., 2010). Inflammasomes activate pro-caspase1, which proteolytically cleaves IL-1β (Netea et al., 2015) IL-1β is powerful pro-inflammatory cytokine shown to exacerbate damage during chronic disease and acute tissue injuries (Dinarello, 2010). Additionally, IL-1β release indicates caspase-1 activation. Caspase-1 can trigger pyroptosis, which is similar to apoptosis, but does not require the activation of death caspases (Brennan and Cookson, 2000; Jesenberger et al., 2000). Therefore, it could be suggested that IL-1β activation by PRVABC59 ZIKV could lead to a serious clinical outcome due to severe inflammation and pyroptotic cell death. The importance of this discovery is that PRVABC59 ZIKV, South American strain, belong to a group of ZIKV associated with newborn microcephaly, while IBH30656 ZIKV strain (African origin) has not been linked to microcephaly. We believe that severe damage to fetal brain tissue and consequent microcephaly could be the result of ZIKV-caused inflammasome activation by the South American strains of virus. We have shown that IL-1β is activated in HUVECs infected with both ZIKV strains, suggesting that the pathogenesis of ZIKV-caused microcephaly is complex, where crossing the placenta and targeting neuronal progenitors are essential. It could be suggested that these features are characteristic only for the South American ZIKV, while they are absent in African strains.

IL-15, which promotes proliferation of natural killer cells (NKs) and plays important role in innate antiviral defense (Biron et al., 1999), was downregulated in ZIKV-infected endothelial cells. IL-15 induces anti-viral defense by an activation of IFN-α production (Foong et al., 2009) and stimulation of NK activity (Azimi et al., 2000; Ashkar and Rosenthal, 2003). The mechanisms of IL-15 downregulation remain to be determined; however, it could be suggested that decreased interleukin production may contribute to virus replication.

We, for the first time, show that ZIKV infection affects MMP expression in endothelial cells. Interestingly, the levels of MMP10 and MMP13 were upregulated in infected HUVECs and the upregulation of MMPs production is commonly seen in dysregulated extracellular matrix leading to inflammation, angiogenesis, and leukocyte trafficking (Page-McCaw et al., 2007). Our most intriguing observation was that ZIKV infection led to a decreased level of MMP8 from the endothelial cells. Recent study by Gutierrez-Fernandez et al. has demonstrated that MMP8 has more complex role in inflammation as its deficiency prolongs inflammation and delays the wound healing (Gutierrez-Fernandez et al., 2007). Another study by Fang et al. (2013) demonstrated the MMP8 knockout mice have less endothelial cell sprouting, migration, and capacity to proliferate, which explains why the lack of this MMP delays wound healing. Increased MMP10 and MMP13 are further supporting the notion that ZIKV infection promotes inflammation and extracellular remodeling. MMP13 is absent in normally healing wounds but is largely expressed in chronic lesions (Toriseva et al., 2012). Also, increased expression of MMP13 was shown to promote inflammation and cartilage destruction (Singh et al., 2013). Our hypothesis for MMP’s role in ZIKV-caused HUVEC monolayer leakage is supported by the data collected using MMP inhibitors. We showed that MMP inhibition restores the permeability of endothelial monolayer. Together, these data support the role of MMPs in ZIKV-induced inflammation and tissue destruction.

Our data demonstrate that ZIKV compromises endothelial monolayer permeability. Interestingly, increased permeability was found in HUVECs infected with both PRVABC59 and IBH30656 strains of ZIKV. These results contrast the previous findings showing the lack of changes in ZIKV-infected HBMECs monolayer permeability (Papa et al., 2017). The variation in endothelial permeability could be related to the physiological differences between HBMECs and HUVECs. It has been shown that, under stress, HBMECs can retain cobbled stone form and minimize the span of tight junctions per unit of the capillary length, which is essential to reduce permeability (Ye et al., 2014). It appears that HUVECs obtain spindle shape, which can increase the monolayer’s permeability. Our findings corroborate the data recently published by Szaba et al. (2018), where embryonic endothelial cell necrosis was demonstrated in ZIKV-infected fetus. The compromised permeability of HUVECs could also be associated with ZIKV-caused cell damage.

We found that increased ZIKV permeability was associated with decreased VE cadherin expression and increased MMP expression. VE cadherin is found exclusively in endothelial cells, where it regulates expression of multiple junction molecules and important adhesion molecules (Taddei et al., 2008; Giampietro et al., 2012; Ye et al., 2014). Interestingly, it appears that low VE cadherin expression is associated with upregulation of MMPs (Llorens et al., 1998). This reciprocal expression of VE cadherin and MMPs is essential for regulation of endothelial cell growth, angiogenesis, and permeability (Kiran et al., 2011). We suggest that downregulation of VE cadherin in ZIKV-infected cells could be related to upregulation of MMPs.

This assumption is supported by our observation of an increased expression of an active form of caspase-8. Caspase-8 is an initiating caspase, which is activated by proteolytic processing in the cytoplasm (Salvesen and Dixit, 1999). Caspase-8 can be activated by the death receptors of the Fas/tumor necrosis factor receptor family (Kischkel et al., 1995). Once activated, caspase-8 can propagate the apoptotic signal by proteolytically releasing downstream executing caspases or by cleaving the BH3-interacting domain death agonist (BID), which migrates to mitochondria and triggers the release of cytochrome c (Luo et al., 1998). Once released, cytochrome c activates caspase-9, which plays a role in the execution phase of apoptosis (Juin et al., 1999). Interestingly, the pathway analysis revealed cytochrome c release in ZIKV-infected HUVECs; therefore, we suggest that ZIKV-caused endothelial cell apoptosis is due to caspase-8-induced cytochrome c release.

In conclusion, our data for the first time demonstrate that ZIKV triggers changes in transcription of genes involved in multiple cellular pathways. Also, the transcriptional activation was detected early following the infection with significantly more genes due to South American strain as compared to African strain. Changes in the cytokine and MMP levels by ZIKV could explain inflammation and tissue destruction caused by infection. Additionally, activated cytokines could contribute to monocyte differentiation and migration across the endothelium tissue barrier.

## Author Contributions

SK and TU performed the experiments. KK and EG performed the pathway analysis. SJ performed the sequencing. AR analyzed the data. SK and SV analyzed the data and wrote the manuscript.

## Funding

RAA was supported by Program of Competitive Growth of Kazan Federal University and state assignment 20.5175.2017/6.7 of the Ministry of Science and Higher Education. This publication was made possible by a grant from the National Institute of General Medical Sciences (GM103440) from the National Institutes of Health. This work was supported by the institutional and departmental funds.

## Conflict of Interest Statement

SJ was employed by Genequest LLC, Reno, NV.

The remaining authors declare that the research was conducted in the absence of any commercial or financial relationships that could be construed as a potential conflict of interest.
